# Sir John Tomes

**Published:** 1895-10

**Authors:** 


### SIR JOHN TOMES.

The death of this distinguished man has created a deep impression throughout the world of scientific thought. It can be said with truth that no man connected with dentistry has so marked his individuality upon it as the subject of this sketch, and, perhaps, no one has done more to place the dental profession on a solid scientific foundation.

He was born at Weston-on-Avon in 1815, and studied at Kings College and the Middlesex Hospitals. He commenced practice in 1840.

His first work was published in 1848, entitled "Dental Physiology and Surgery." He says, in the preface of this, "When I had the honor to accept the office of dentist to the Middlesex Hospital, I promised the medical officers that . . . I would deliver a course of lectures on dental physiology and surgery. . . . The first six lectures are devoted to dental physiology, and they contain, I think, some new views on the development of dentine and enamel. . . . These lectures were written for and delivered to beginners, . . . and were not written for those who have learned, but for those who have yet to learn."

The modesty of this concluding paragraph, while characteristic, was unnecessary then as it has been in succeeding years, for these have but deepened the conviction that this work of Sir John Tomes is one of the most valuable ever published upon dental subjects. Future investigators have added but little to his discovery of the dental fibril, which came at a later period, being announced in *Philosophical Transactions*, vol. cxlvi., and subsequently reproduced in his "Dental Surgery," issued in 1859. This latter book is still used as a text-book; in fact, nothing that Sir John has written but has a distinct value, not in the least diminished by years.

He led the way in the investigation of the minute anatomy of tooth-structure, and shed such a flood of light upon the histology of the tissues connected therewith that the first period of investigations in this direction may be said to have practically ended with his labors.

He aided materially in the improvement of extracting-forceps, basing their formation on true anatomical considerations.

His name, however, will go down into professional history for his scientific work, which was continued to a late period of his active life.

He was an energetic participant in the long fight in England to advance the dental profession in a legal and educational sense. He was prominent in the parliamentary warfare that led to the Act of 1878, which made training, diplomas, and registration compulsory.

He retired from practice some years since, and has resided at Caterham.

He was knighted in 1886, in recognition of "eminent services rendered his profession."

His death will be sincerely mourned on this side of the Atlantic; but while all must feel that the world has lost one of its ablest workers, there remains still the compensating thought that he lived to see the seeds which he planted in his early professional life, bearing rich fruitage for the advancement of his chosen profession throughout the world.

